# Mitigating food safety risks in Indonesia's free school meals programme

**DOI:** 10.1016/j.lansea.2026.100734

**Published:** 2026-02-18

**Authors:** Henry Surendra, Arif Sujagat, Grace Wangge

**Affiliations:** a Public Health Program, Monash University Indonesia, Tangerang, Indonesia; b Oxford University Clinical Research Unit Indonesia, Faculty of Medicine, Universitas Indonesia, Jakarta, Indonesia; c National Centre for Epidemiology and Population Health, Australian National University, Canberra, Australia

Globally, universal free school meals have been reported to potentially solve issues on food insecurity and nutrition deficiencies for students.^1^ In the US, school meals may provide healthier options than meals brought from home, and benefit students academically in the long term, by improving meaningful school attendance.^1^ In lower-resource settings such as Brazil and India, the free school meals have shown several benefits, including improved nutritional status, support for more sustainable food systems, attendance and academic performance.^2^^,^^3^

In January 2025, the Indonesian government launched a national free nutritious meals programme to improve nutrition and health outcomes for 82.9 million beneficiaries. The programme is expected to support government efforts in addressing Indonesia's triple burden of nutrition.^4^ As per 19 December 2025, more than 46 million school children received the free meals.^5^ However, government report indicated an increasing number of food poisoning cases among school children receiving the free meals.^6^

We reviewed existing policies, guidelines and official government news reports, and interviewed surveillance officer to collect data on total numbers of food poisoning cases, general symptoms, date and location of outbreaks, and further information on the findings from outbreak investigations conducted across Indonesia. Our analysis reveals that as per 31 December 2025, the free school meals programme has caused 177 food poisoning outbreaks, affecting the health of more than 20,000 schoolchildren across 127 districts in 33 of 38 provinces in Indonesia (Supplementary Figure S1). The true magnitude of the outbreaks is more likely to be much higher than reported. The reported symptoms ranged from diarrhoea, stomach pain, nauseas, vomiting, fever, seizure and shortness of breath. Cause of outbreaks were not confirmed for most of the outbreaks. Several laboratory investigations confirmed that contamination by biological agents such as *E. coli*, *Campylobacter*, *Salmonella*, or *Staphylococcus* caused the food poisoning outbreaks. Case detection and mitigation efforts on the ground are currently lacking, as the local district health offices were not involved in the implementation and monitoring of the programme. Specific official guidelines on how to conduct surveillance, outbreak detection and response, are currently non-existent.

Additionally, programme implementation faces major challenges. Despite being established just four months before programme rollout, Indonesia's National Nutrition Agency (*Badan Gizi Nasional*–BGN) was mandated to rapidly scale-up implementation through newly formed nutritional provision service units (*Satuan Pelaksana Pemenuhan Gizi*–SPPG). Each unit is typically required to provide 3000–4000 portions of meals daily for 15–25 local schools, in addition to monthly contributions to 5–10 community health posts. Operations follow a demanding daily schedule including overnight cooking. The BGN's design—centralising authority without documented readiness assessments—burdened frontline SPPG, likely contributing to the surge of food poisoning outbreaks.

Based on official government report, of 11,592 units, only 198 have obtained hygiene certificate, and 26 met international standard such as Hazard Analysis and Critical Control Point (HACCP).^7^ Our interview with 162 surveillance officers revealed that many food handlers prepare food without proper protective equipment and handwashing procedures. Raw meat, chicken, seafood and other high-risk ingredients were stored not between safe temperature ranges, enabling bacteria or viruses to proliferate before cooking. Large portions of cooked meals were frequently left at room temperature for 7–8 h before consumption, far exceeding the WHO's 4-h safety threshold. Food trays were likely contaminated when dried with unsterile cloth. Fresh fruits were inadequately rinsed, while utensils and kitchen surfaces were only wiped rather than sterilised. In some food providers facilities, weak sanitation has allowed flies and even maggots to contaminated meals in some cases.

Embedding food safety principles across the food supply chain from procurement, storage, cooking, distribution and consumption is essential in Indonesia's ambition to reduce the burden of malnutrition. Close monitoring on the compliance with HACCP and five keys to safer food principles should become mandatory. Enforcement of time–temperature controls, as recommended by the FAO, WHO and Codex Alimentarius, is critical. Meals that exceed safe storage limits must be discarded rather than served. Finally, investment is needed to ensure appropriate cold storage, freezers, sterilisation equipment, and safe kitchen facilities are in place.

Whilst the free nutritious meals programme offers potential solutions for improving nutrition and health outcomes for vulnerable communities, implementing the programme in limited-resource settings such as Indonesia, required proper planning and close monitoring and evaluation. Implementing similar centralised kitchen systems, India's Mid-day Meal Scheme and Brazil's National School Feeding Programme illustrated the importance of implementing strict regulatory frameworks, adherence to hygienic food preparation practices and local procurement oversight through school nutrition councils. Cross-sectoral involvement and establishment of school-based surveillance and outbreaks response will be essential to mitigate risk of food poisoning in the future. Operational research identifying implementation bottleneck and cost-benefit analysis are needed to support sustainability of the programme.

## Contributors

**HS:** Conceptualization, Data analysis, Data interpretation, Supervision, Writing original draft, Review and editing. **AS:** Data curation**,** Figures**,** Data analysis**,** Data interpretation**,** Writing original draft, Review and editing. **GW:** Supervision**,** Data interpretation, Writing original draft, Review and editing.

All authors have read and approved the final version of this manuscript.

## Editor note

The Lancet Group takes a neutral position with respect to territorial claims in published maps and institutional affiliations.

## Declaration of interests

All authors disclose no competing interest.
