# Supplementary material for: Mitigating food safety risks in Indonesia's free school meals programme
Source: Lancet Reg Health Southeast Asia. 2026 Feb 18;46:100734. doi: 10.1016/j.lansea.2026.100734 (PMC12933291; doi:10.1016/j.lansea.2026.100734)
Supplement: Supplementary Figures S1 [file mmc1.pptx]

## Slide 1
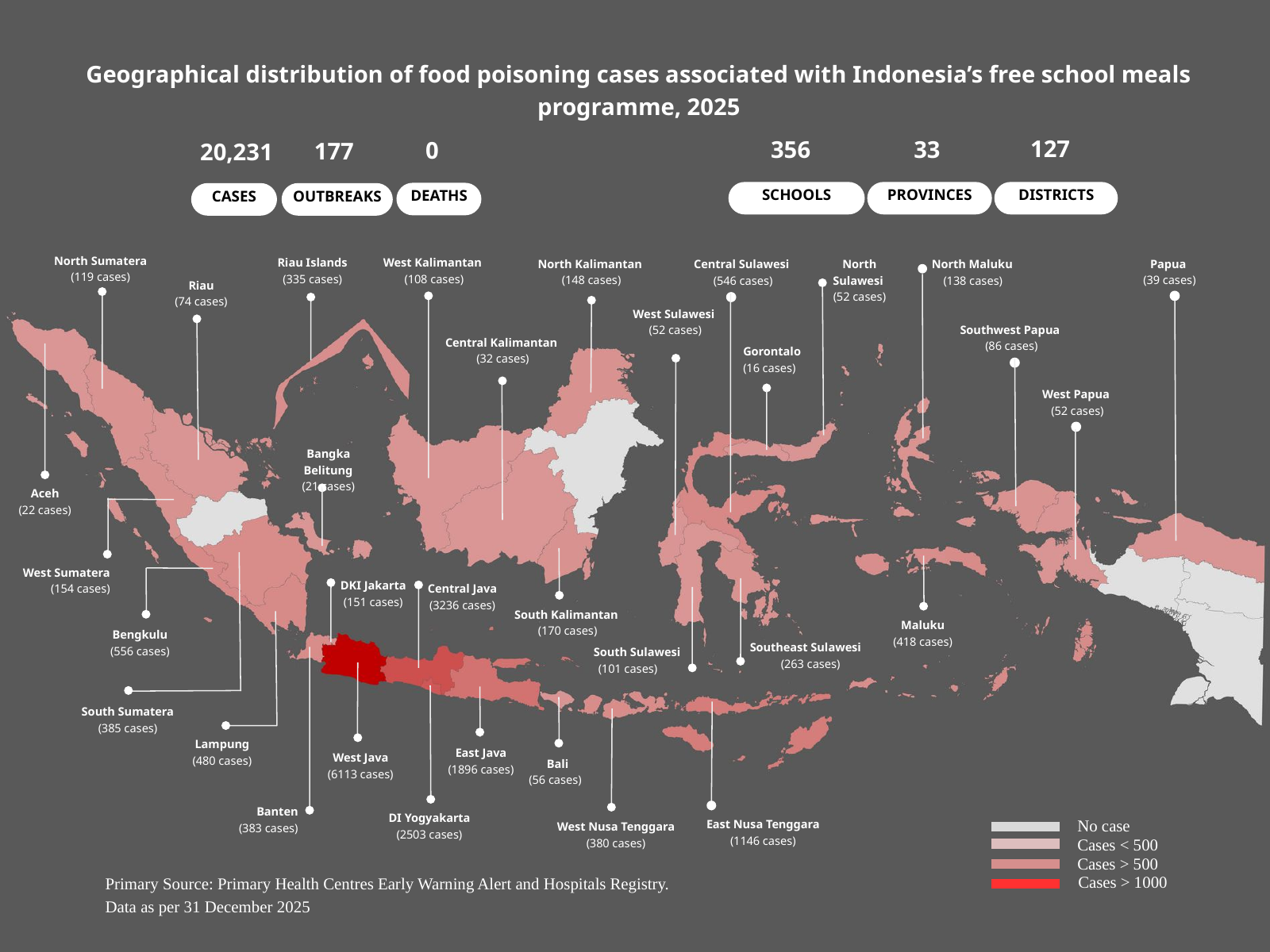

Geographical distribution of food poisoning cases associated with Indonesia’s free school meals programme, 2025
127
0
356
33
177
20,231
SCHOOLS
PROVINCES
DISTRICTS
DEATHS
CASES
OUTBREAKS
North Sumatera
(119 cases)
Riau Islands
(335 cases)
West Kalimantan
(108 cases)
North Kalimantan
(148 cases)
Papua
(39 cases)
Central Sulawesi
(546 cases)
North Sulawesi
(52 cases)
North Maluku
(138 cases)
Riau
(74 cases)
West Sulawesi
(52 cases)
Southwest Papua
(86 cases)
Central Kalimantan
(32 cases)
Gorontalo
(16 cases)
West Papua
(52 cases)
Bangka Belitung
(21 cases)
Aceh
(22 cases)
West Sumatera
(154 cases)
DKI Jakarta
(151 cases)
Central Java
(3236 cases)
South Kalimantan
(170 cases)
Maluku
(418 cases)
Bengkulu
(556 cases)
Southeast Sulawesi
(263 cases)
South Sulawesi
(101 cases)
South Sumatera
(385 cases)
Lampung
(480 cases)
East Java
(1896 cases)
West Java
(6113 cases)
Bali
(56 cases)
Banten
(383 cases)
DI Yogyakarta
(2503 cases)
No case
East Nusa Tenggara
(1146 cases)
West Nusa Tenggara
(380 cases)
Cases < 500
Cases > 500
Cases > 1000
Primary Source: Primary Health Centres Early Warning Alert and Hospitals Registry.
Data as per 31 December 2025
